# Characterisation of four hotdog-fold thioesterases for their implementation in a novel organic acid production system

**DOI:** 10.1007/s00253-020-10519-w

**Published:** 2020-03-19

**Authors:** T. W. P. Hickman, D. Baud, L. Benhamou, H. C. Hailes, J. M. Ward

**Affiliations:** 1 Department of Biochemical Engineering, University College London, Gower Street, London, WC1E 6BT UK; 2 Department of Chemistry, University College London, 20 Gordon Street, London, WC1H 0AJ UK

**Keywords:** Thioesterase, CoA, RpaL, Promiscuous, Organic acid

## Abstract

With increasing interest in the diverse properties of organic acids and their application in synthetic pathways, developing biological tools for producing known and novel organic acids would be very valuable. In such a system, organic acids may be activated as coenzyme A (CoA) esters, then modified by CoA-dependent enzymes, followed by CoA liberation by a broad-acting thioesterase. This study has focused on the identification of suitable thioesterases (TE) for utilisation in such a pathway. Four recombinant hotdog-fold TEs were screened with a range of CoA esters in order to identify a highly active, broad spectrum TE. The TesB-like TE, RpaL, from *Rhodopseudomonas palustris* was found to be able to use aromatic, alicyclic and both long and short aliphatic CoA esters. Size exclusion chromatography, revealed RpaL to be a monomer of fused hotdog domains, in contrast to the complex quaternary structures found with similar TesB-like TEs. Nonetheless, sequence alignments showed a conserved catalytic triad despite the variation in quaternary arrangement. Kinetic analysis revealed a preference towards short-branched chain CoA esters with the highest specificity towards DL-β-hydroxybutyryl CoA (1.6 × 10^4^ M^−1^ s^−1^), which was found to decrease as the acyl chain became longer and more functionalised. Substrate inhibition was observed with the fatty acyl *n*-heptadecanoyl CoA at concentrations exceeding 0.3 mM; however, this was attributed to its micellar aggregation properties. As a result of the broad activity observed with RpaL, it is a strong candidate for implementation in CoA ester pathways to generate modified or novel organic acids.

**Electronic supplementary material:**

The online version of this article (10.1007/s00253-020-10519-w) contains supplementary material, which is available to authorized users.

## Introduction

Over the last century, various organic acids have been produced at an industrial scale due to their uses as bulk and specialty chemicals. However, in recent years, the commercial range of organic acids has been dramatically expanded due to intensive academic and industrial research into novel organic acids. As a result of this expansion, current applications include cosmetics, preservatives, antimicrobials, polymers and pharmaceuticals (Van Immerseel et al. 2006; Alonso et al. 2015; Zhitnitsky et al. 2017). With the chemical portfolio of organic acids increasing, the global production of these compounds has increased accordingly. However, for many of these compounds, the mainstay production method is through the use of petroleum-derived substrates, which are both finite and environmentally detrimental. In response, there has been a recent surge in research focused on the microbial fermentative production of organic acids, which has led to the industrial scale production of a number of organic acids through fermentation (Carlson et al. 2016). It is thus clear that the design and construction of metabolic systems for the production of existing and novel organic acids is, and will continually become, an extremely useful tool.

**Fig. 1:**
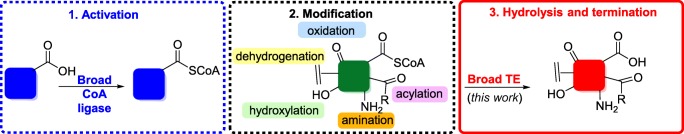
The 3 modules of the proposed biosynthetic CoA ester modification system. Module 1 (activation) will utilise a broad spectrum CoA ligase to activate a range of organic acids into their corresponding CoA esters. Module 2 (modification) will add bespoke functional complexity to the CoA ester using enzymes from a developed toolbox of CoA-dependent enzymes. Module 3 (termination) will utilise a broad spectrum TE to hydrolyse a range of CoA esters into their corresponding organic acids. Dashed boxes indicate ongoing work and the solid line indicates work presented in the current investigation

The synthesis of coenzyme A (CoA) thioesters through the activation of organic acids is an essential cellular process found in all living organisms. Once converted into their corresponding thioesters, the CoA-bound organic acid is targeted towards a plethora of enzyme cascades within both primary and secondary metabolism. Here, CoA esters can either be directly modified or covalently transferred to acyl carrier protein (ACP) domains of multi-subunit enzymes, such as polyketide synthases and fatty acid synthases. The bound organic acid can then undergo a range of biotransformations including acyl transfer, Claisen condensation, Michael addition and β-elimination procedures (Mishra and Drueckhammer 2000). The organic acid component of intermediates from these cascades are often novel compounds, having been modified in a stereo- and regiospecific manner that could not easily be achieved through conventional chemical synthesis. With the need for synthesising more complex organic acids to be used as synthons for specialty compounds, the utility of CoA ester-modifying biological cascades would be an innovative approach for producing a plethora of novel organic acids. With this understanding, a hypothetical biological system for producing novel organic acids has been proposed (Fig. 1).

The first module (activation) of this system would utilise the activity of a broad acting CoA ligase, which would produce CoA esters from a range of organic acid substrates. In the second module (modification), a number of CoA-dependent enzymes and biological modules can be used, providing highly specific functional complexity to the CoA ester. Finally, the third component (termination) of this system would employ a broad spectrum thioesterase (TE) to hydrolyse the thioester bond, releasing the novel complex organic acid. The importance of the CoA ligase and TE having broad activity would mean they can be used with a plethora of substrates and products. Moreover, the development of a ‘toolbox’ of CoA-dependent enzymes would facilitate a ‘plug and play’ approach to these enzymes in order to achieve the desired complex organic acid. The current study focuses on the final aspect of this system, which requires a broad acting TE.

TEs (EC 3.1.2.1-27) are a diverse and widespread class of enzyme that are categorized into superfamilies I and II, which are subcategorized into 25 families based on their primary and tertiary structures on the ThYme (Thioester-active enzYmes) database (Gonzalez et al. 2012). Superfamily I consist of α/β-hydrolase-fold TEs, and superfamily II share a common ‘hotdog’ fold, so named due to a seven-stranded antiparallel β-sheet, referred to as the ‘bun’, being wrapped around a five-turn α-helix ‘sausage’ (Dillon and Bateman 2004). Both classes of TEs function by catalysing the hydrolysis of thioesters; however, members of family I are commonly a component of a multi-subunit domain protein such as a polyketide synthases (PKS), whereas superfamily II are commonly independent enzymes (Lenfant et al. 2013). Furthermore, superfamily II have been previously shown to target both CoA and ACP thioesters with promiscuous activity, suggesting enzymes within this superfamily to be good candidates for our intended purpose.

The hotdog-fold domain was initially identified within FabA from *Escherichia coli* and subsequently in 4-hydroxybenzoyl-CoA TE from *Pseudomonas* sp. strain CBS. Despite TEs within this superfamily having a conserved hotdog fold, a lot of variation has been found within both the tertiary and quaternary structures. The tertiary structure of these TEs has been shown to range from hotdog-fold monomers to fusion proteins containing two tandem hotdog-fold domains, as well as hotdog-fold domains linked to domains with different functions (Dillon and Bateman 2004). Furthermore, the crystal structures of hotdog-fold TEs resolved to date show a plethora of different quaternary structures, including hotdog-fusion monomers, homodimers (front to front and back to back), tetramers and hexamers (Pidugu et al. 2009).

The aim of this investigation was to isolate a number of TEs from superfamily II and screen their activity with a broad range of aliphatic and aromatic CoA esters. Then, based on the screening results, determine a TE that would be a suitable candidate for future implementation within the proposed CoA ester modification system. Four TEs were isolated, three members of the 4-HB-CoA subfamily: FcbC (*Arthrobacter* sp. Strain AU, GenBank accession MN428870), which has been previously shown to have activity towards short and medium chain aliphatic CoA esters, aromatic CoA esters with various hydroxyl groups, as well as dihydroxylated and chlorinated aromatics (Song et al. 2012), PA2801 (*Pseudomonas aeruginosa* PAO1, GenBank accession MN428871) which has been shown to have activity towards a range of aliphatic CoA esters ranging from acetyl CoA to palmitoyl CoA (Gonzalez et al. 2012), and YbdB (*E. coli* K-12 MG1655, GenBank accession MN428873), which functions as a 2,3-dihydroxybenzoyl-*holo*EntB proofreader in the biosynthesis of the siderophore enterobactin and has been shown to have inherent promiscuity, with a bias towards aryl-CoAs (Latham et al. 2014; Wu et al. 2014). In addition, an uncharacterised member of the TesB-like subfamily, RpaL (GenBank accession MN428872) from *Rhodopseudomonas palustris* HaA2.

## Materials and methods

### Strains and cultures

*Escherichia coli* TOP10 was used for storage and replication of recombinant plasmids. For protein expression, plasmids were extracted using the Qiagen miniprep kit and transformed into *E. coli* BL21 (DE3). All *E. coli* strains were grown in Lysogeny broth (LB) media and incubated at 37 °C with 50 μg/ml Kanamycin, shaking at 250 rpm, unless otherwise stated. For growth on solid media, LB agar was used. For plasmid extractions, starter cultures and glycerol stocks, 10-ml cultures were grown overnight in 50-ml falcon tubes containing appropriate antibiotic. For protein expression, 1 ml of a starter culture was added to 49 ml of LB in 250-ml conical flasks and grown to an optical density of 0.5–0.8 OD_600_ (exponential growth), at which point cultures were induced with 1 mM isopropyl-β-D-galactopyranoside (IPTG). Following 6 h of IPTG-induced expression at 37 °C cultures were transferred into 50-ml falcon tubes, pelleted and stored at − 20 °C.

### DNA synthesis and cloning procedures

pET29a was used as the primary cloning and expression vector for all TEs. The exceptions to this were FcbC, which was synthesised by DNA2.0 into pD451-SR: an IPTG inducible vector and RpaL from *Rhodopseudomonas palustris* HaA2 which was synthesised by Eurofins (Luxemburg) and sub-cloned into pET29a using synthetic restriction sites *Nde*I and *Xho*I. RpaL and FcbC were codon-optimised for expression in *E. coli*, using the sequence manipulation suite online tool (available at: www.bioinformatics.org/sms2/rev_trans.html). The accession number for the codon-optimised FcbC gene is MN428870 and for the codon-optimized RpaL is MN428872.

PA2801 and YbdB were amplified from the reference genomes: *Pseudomonas aeruginosa* PAO1 (NC_002516.2) and *E.coli* K12 MG1655 (NC_000913.3), respectively. For the identification of PA2801, the characterised protein (accession: 3QY3_A) (Gonzalez et al. 2012) was used to identify the nucleotide sequence (Gene ID: 879843) within the *P. aeruginosa* PAO1 reference genome. For identification of YbdB, the previously characterised enzyme (accession: P0A8Y8) was used to search the reference genome (NC_000913.3) leading to the identification of the nucleotide sequence (Gene ID: 945215) used in this study. For each PCR, 0.02 U/μl Q5 High-Fidelity DNA polymerase (NEB), 200 μM dNTPs, × 1 Q5 reaction buffer and 0.5 μM of each primer was brought to a final volume of 25 μl with Milli-Q water.

The primer sequences for PA2801 and YbdB were as follows:

- PA2801 forward: CGAGGAGAGAATTC**ATG**GCTGACAGACAATTGC
- PA2801 reverse: GCTTTGACTAGT**TCA**GGCGATCGCGGCG
- YbdB forward: GCTAAATTCGAGGAGAGAATTC**ATG**ATCTGGAAACGCCATTTAAC
- YbdB reverse: CTTTCGGGCTTTGACTAGT**TCA**TCCCAAAACTGCCG

PA2801 and YbdB were cloned into pET29a for expression using circular polymerase extension cloning (CPEC), following the methods stated previously (Quan and Tian 2011). Therefore, primers used were also required to introduce overlapping regions between the vector and inserts (underlined), allowing complementary base-pair binding and polymerase extension in the CPEC reaction. Start codons and stop codons have been annotated in bold font.

The pET29a primers for PA2801 were as follows:

- Forward: CGATCGCC**TGA**ACTAGTCAAAGCCCGAAAGGAAG
- Reverse: GTCAGC**CAT**GAATTCTCTCCTCGAATTTAGCAGCAGCGG

The pET29a primers for YbdB were as follows:

- Forward: CGGCAGTTTTGGGA**TGA**ACTAGTCAAAGCCCGAAAG
- Reverse: CCAGAT**CAT**GAATTCTCTCCTCGAATTTAGCAGCAGCGG

### Extraction and quantification of thioesterases

For the extraction of soluble protein, frozen stock pellets of cultures expressing each of the TEs were thawed and lysed. Here, 20 mg of the cell pellet was measured and then re-suspended in 100 μl of Bugbuster protein extraction reagent (Novagen, Inc. 70,584-3). After 15 min of mixing at room temperature, suspensions were centrifuged (20 min, 4 °C, 11,500 rpm) separating soluble protein from the insoluble components of the cell suspension. A Bradford assay was then used to quantify soluble protein by measuring the absorbance at 595 nm of known concentrations of bovine serum albumin (BSA) and producing a standard curve (concentration vs. OD_595_). Once quantified, 20 μg of clarified lysate was then loaded on an SDS gel for confirmation of TE expression. All SDS gels were stained using Instant Blue (Sigma-Aldrich).

### DTNB assay

TE activity was determined by using the 5,5′-dithio-bis(2-nitrobenzoic acid) (DTNB) colorimetric assay (Ellman 1959). In the presence of free thiol groups, DTNB is cleaved and the 2-nitro-5-thiobenzoate (TNB) released can be detected at 405 nm. For 1 mol of thiol, 1 mol of TNB is produced, allowing CoA concentration to be directly inferred from TNB concentration. Assays were run at 25 °C and contained: 20 μg clarified lysate, 100 mM triethanolanime (TEA) buffer (pH 8), 0.2 mM CoA substrate, 0.4 mM DTNB and ultrapure Mili-Q water to a total volume of 250 μl. Reactions were started by the addition of the CoA substrate and run for 30 min. Control reactions using lysate from cultures containing empty pET29a was used to account for any background cleavage of CoA substrates. CoA concentrations determined in control reactions (containing no enzyme) were subtracted from TE reactions for determination of enzymatically hydrolysed CoA. All assays were performed in triplicate in a 96-well plate and monitored using a TECAN Safire^2^ ™ (Invitrogen ™) microplate reader.

### His-tag purification of RpaL

In order to purify RpaL an N-terminal His-tag was added to its open reading frame (ORF). This was done by sub-cloning RpaL into pET28a using the cut sites *Nde*I and *Xho*I*.* The new construct RpaL-His was then transformed into *E. coli* BL21 (DE3) and cultured and expressed as previously stated. Cell pellets were then re-suspended in 10 mM imidazole and lysed through sonication under the conditions: 10 s on, 10 s off for 10 cycles, using a Soniprep 150 (© 2018 MSE Centrifuges). Lysed cultures were then centrifuged followed by the transfer of soluble fraction to a nickel column. Wash steps with increasing imidazole concentrations were used (50 mM and 100 mM) with a final elution concentration of 500 mM. Eluted protein was then stored at 4 °C with 50% (w/v) ammonium sulphate.

### Determination of steady-state kinetic constants of RpaL

The DTNB colorimetric assay was used to determine the production of CoA from the CoA thioesters. The same conditions as previously mentioned were used with the exception of 2 μg (0.73 μM) of purified RpaL rather than clarified lysate being used. Steady-state kinetic measurements for RpaL were determined by measuring the initial rates (*V*_*0*_) of CoA ester hydrolysis as a function of time. Following the addition of a CoA ester, the first time points showing an increase in CoA concentration were used to determine the initial rate (*V*_*0*_). All reactions were run for 30 min; however, the initial rates were determined from time points obtained within the first 5 min. Varying concentrations of each of the 6 CoA esters (*i*Val CoA, Bz CoA, *i*But CoA, HMG CoA, DLBH CoA and *n*HD CoA) were assayed, in triplicate, to create substrate [S] saturation curves. These curves were then fitted to the Michaelis–Menten equation *V*_*0*_ *= V*_max_[*S*]/ ([*S*] + *K*_*m*_), using the Origin 2017 software, allowing determination of maximal velocity (*V*_max_*)* and Michaelis–Menten constant (*K*_m_*)*. Turnover (*K*_cat_) could then be obtained using the equation *K*_cat_ *= V*_max_/[*E*].

### Size exclusion chromatography

Size exclusion chromatography (SEC) was used to determine the biological configuration of purified RpaL. An HPLC 1660 was used with an Agilent Zorbax 250 column. Phosphate-buffered solution (PBS) degassed with helium was used as the mobile phase and run at a flow rate of 0.4 ml/min and a run time of 10 min. Purified RpaL was buffer exchanged after purification into PBS. An injection volume of 25 μl was used.

### CoA calibration curve

In order to calculate the concentration of CoA produced, a standard curve was created using known concentrations of CoA. Each standard was measured at 405 nm and contained 0.4 mM DTNB, 100 mM of TEA and water, to a total volume of 250 μl. Once set up, but prior to measuring at 405 nm, these standards were mixed and incubate for 5 mins at 25 °C.

### Chemicals

All CoA ester substrates were bought from Sigma-Aldrich with the exception of cyclohexanecarboxylic acid coenzyme A (CHC CoA) and *S*-(2-acetamidoethyl) cyclohexanecarbothioate (CHC-NAC). These two substrates were chemically synthesised according to methods stated in supplementary material (Figure S1–3).

## Results

### Cloning and expression of four thioesterases

**Fig. 2:**
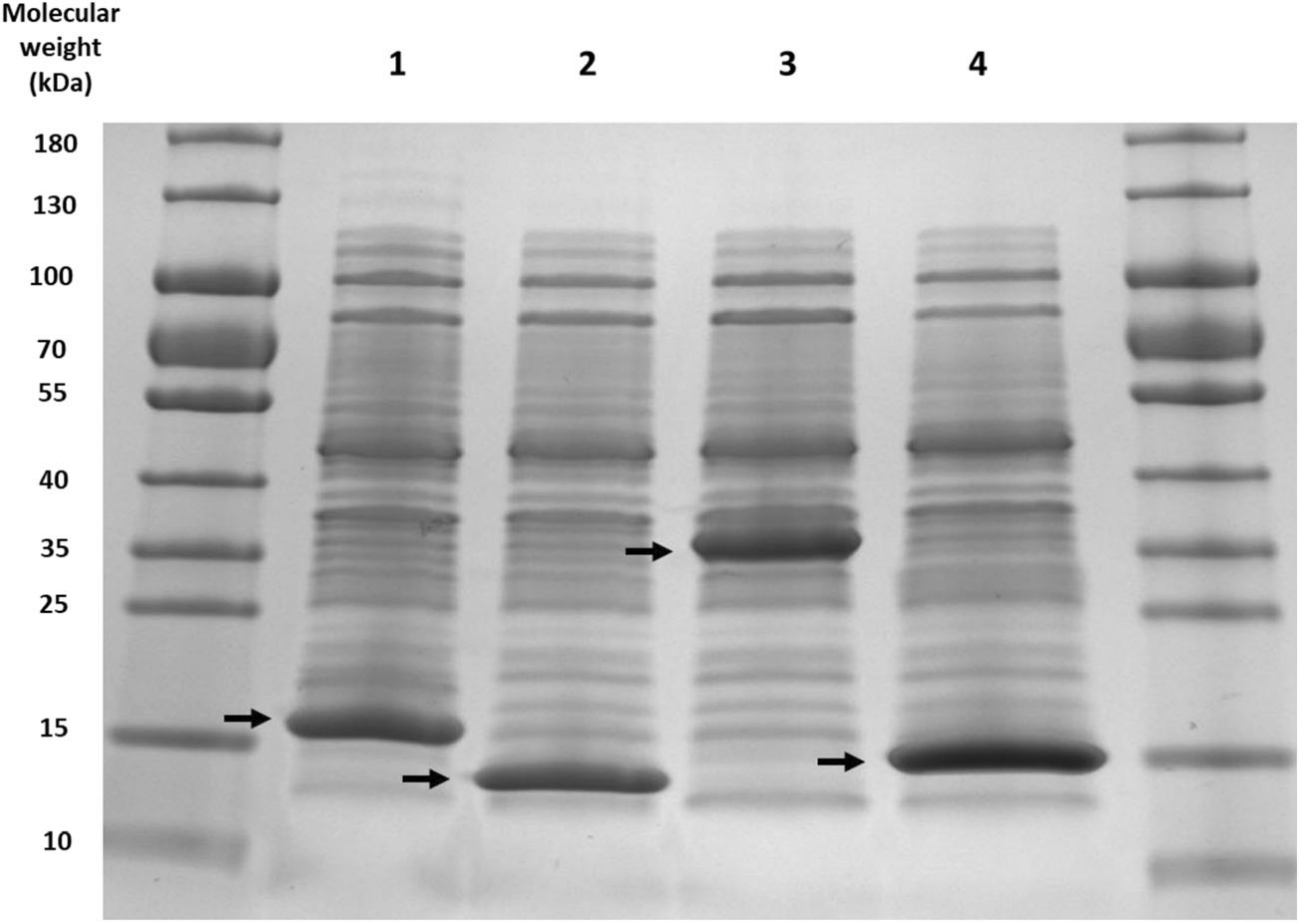
An SDS gel containing the clarified lysate from BL21 (DE3) cells expressing (1) FcbC (17.4 kDa), (2) PA2801 (14.9 kDa), (3) RpaL (33.3 kDa) and (4) YbdB (14.9 kDa) (highlighted by black arrows). For each sample, 10 μg of total protein was loaded to each well. Samples were run alongside 5 μl of PageRuler™ prestained protein ladder for size determination

The model candidate TE for this CoA ester modification system would have high activity towards a range of CoA esters (i.e. long- and short-chain aliphatics, aromatic and alicyclic CoA esters). Four TEs were chosen, three of which had previous characterisation (FcbC, PA2801 and YbdB) and the fourth was an uncharacterised TE (RpaL) from *Rhodopseudomonas palustris* (HaA2). Plasmids (pET29a) containing each of the four TEs were used to transform *E. coli* BL21 (DE3) cells, which were then assessed for soluble protein expression. The SDS gel results showed all four TEs to be expressed within the soluble fraction of lysed *E. coli* BL21 (DE3) cells. The size of each TE monomer was FcbC, 17.4 kDa; PA2801, 14.9 kDa; RpaL, 33.3 kDa; and YbdB, 14.9 kDa (Fig. 2).

### Substrate screening of thioesterases

**Fig. 3:**
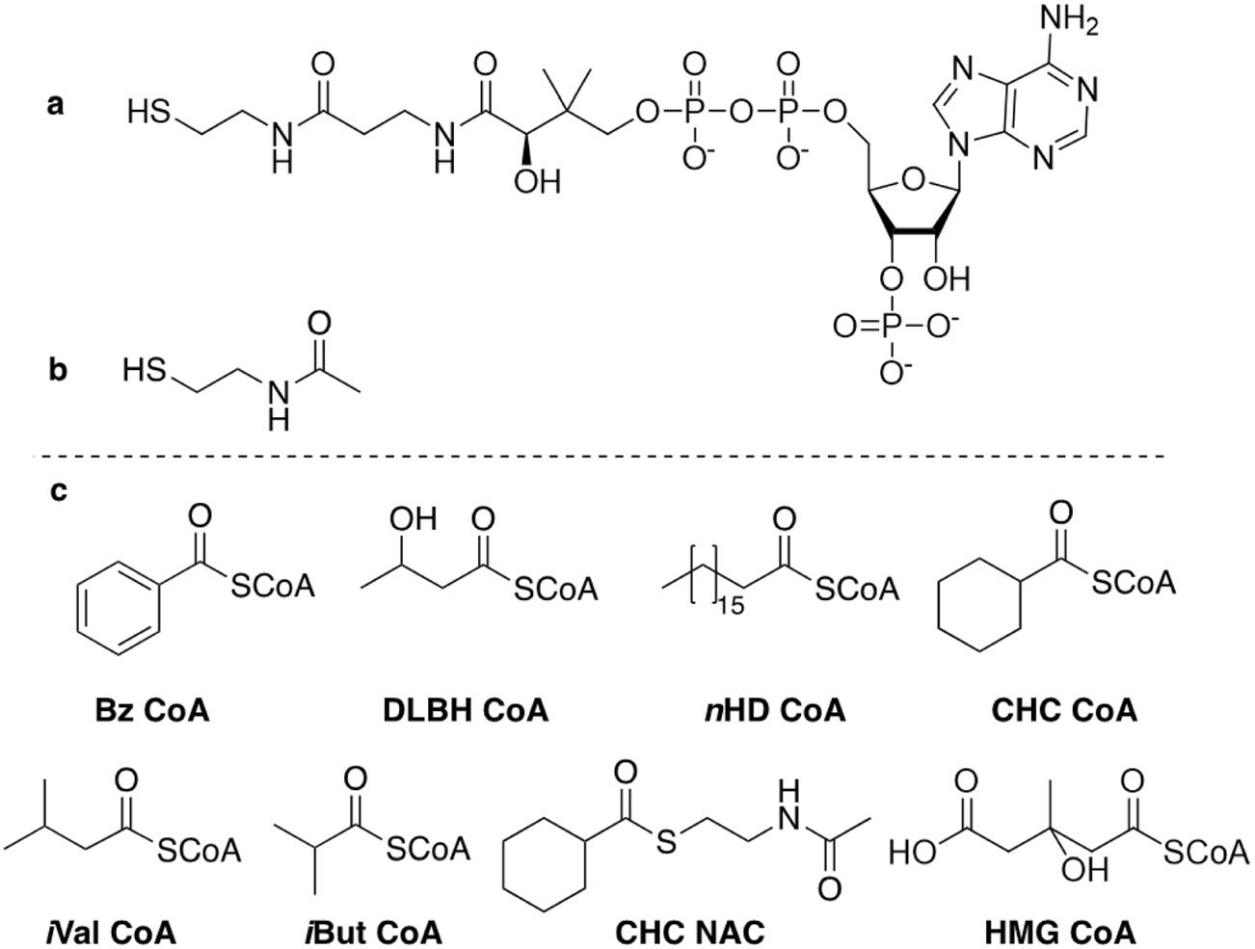
The chemical structure of **a** coenzyme A, **b***N*-acetylcysteamine and **c** all thioesters used as substrates for TE screening: benzoyl CoA (Bz CoA), DL-β-hydroxybutyryl CoA (DLBH CoA), *n*-heptadecanoyl CoA (*n*HD CoA), cyclohexanecarbonyl-CoA (CHC CoA), isovaleryl CoA (*i*Val CoA), isobutyryl CoA (*i*But CoA), cyclohexanecarbonyl N-acetylcysteamine (CHC-NAC) and β-hydroxy β-methylglutaryl CoA (HMG CoA)

**Fig. 4:**
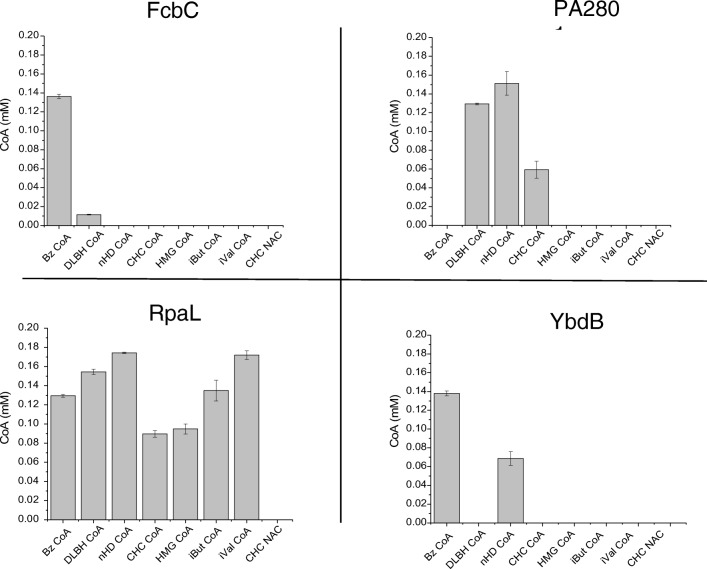
The activity of each of the four recombinant TEs, as part of a clarified lysate, with a range of CoA esters and the synthetic CHC CoA mimic—CHC-NAC. The TEs were assayed using the DTNB assay, which in the presence of a free thiol group (CoA) cleaves, releasing TNB, which is monitored at 405 nm

The hydrolytic activity of each TE was determined with a number of CoA esters (Fig. 3c). FcbC was found to have activity towards benzoyl CoA (Bz CoA) and the aliphatic substrate DL-β-hydroxybutyryl CoA (DLBH CoA) (Fig. 4). This result aligned with previous investigations which had looked into the activity of FcbC, and showed it to have activity towards a range of aromatic CoA esters, yet comparably little activity towards aliphatic CoA esters (Zhuang et al. 2003). PA2801 was found to have activity towards the aliphatic CoA esters: DLBH CoA, *n*-heptadecanoyl CoA (*n*HD CoA) and the cyclic CoA ester, CHC CoA; however, no activity was observed with Bz CoA or either of the branched chain CoA esters, isovaleryl CoA (*i*Val CoA) and isobutyryl CoA (*i*But CoA). Interestingly, despite activity being found with CHC CoA, no activity was found with CHC-NAC. RpaL was found to have activity towards all the CoA esters that were screened, including both aliphatic and aromatic CoA esters. RpaL, like all four TEs examined here, was unable to use CHC-NAC despite having activity towards CHC CoA, suggesting hotdog-fold TEs require the entire CoA to facilitate hydrolysis. YbdB was found to only have activity towards Bz CoA and *n*HD CoA, which aligned with a previous investigation showing it to have a preference towards aromatic and long-chain fatty acyl-CoA esters, with no activity towards short-branched chain CoA esters such as β-methylcrotonyl-CoA (Latham et al. 2014).

### RpaL purification and size exclusion

**Fig. 5:**
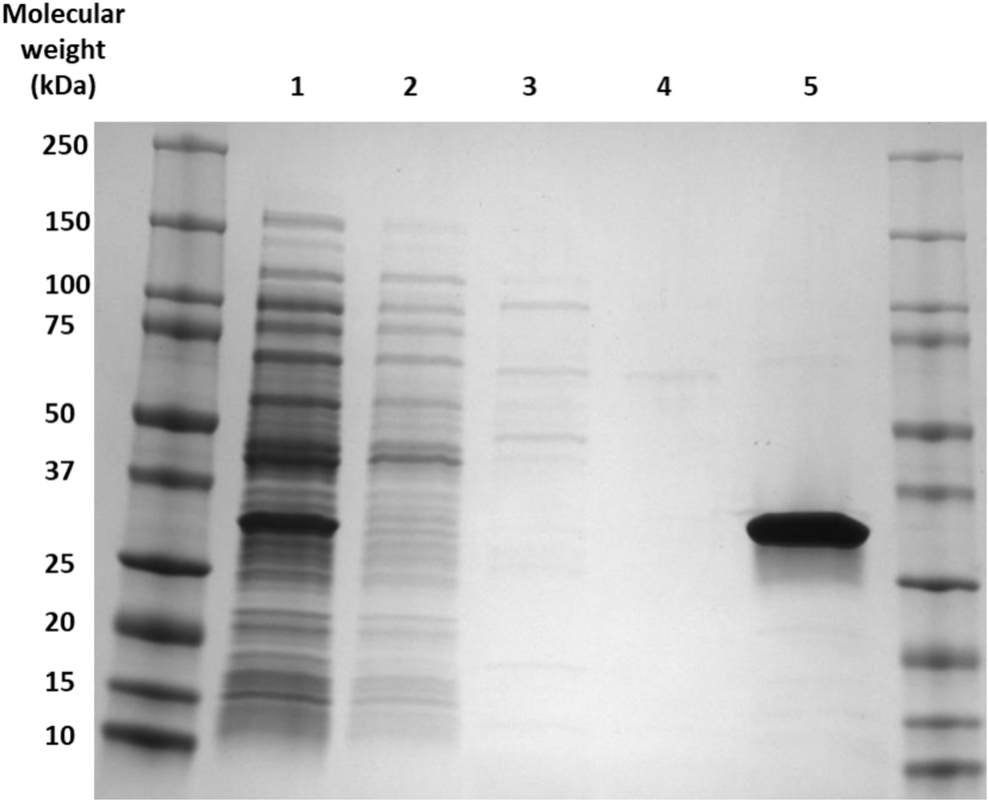
An SDS gel showing the His-tag purification of RpaL from soluble lysate. Lane 1 shows RpaL expressed within the soluble lysate. Lanes 2, 3 and 4 show protein eluted within the wash steps, containing 10 mM, 50 mM and 100 mM imidazole, respectively. Lane 5 shows purified RpaL eluted with 500 mM imidazole

As a result of the broad substrate specificity found within the clarified lysate of cells expressing RpaL, it was sub-cloned into pET28a, attaching an N-terminal His-tag for nickel column purification. Lysate-containing expressed RpaL was loaded onto a nickel column and RpaL was purified to homogeneity with 500 mM imidazole (Fig. 5).

**Fig. 6:**
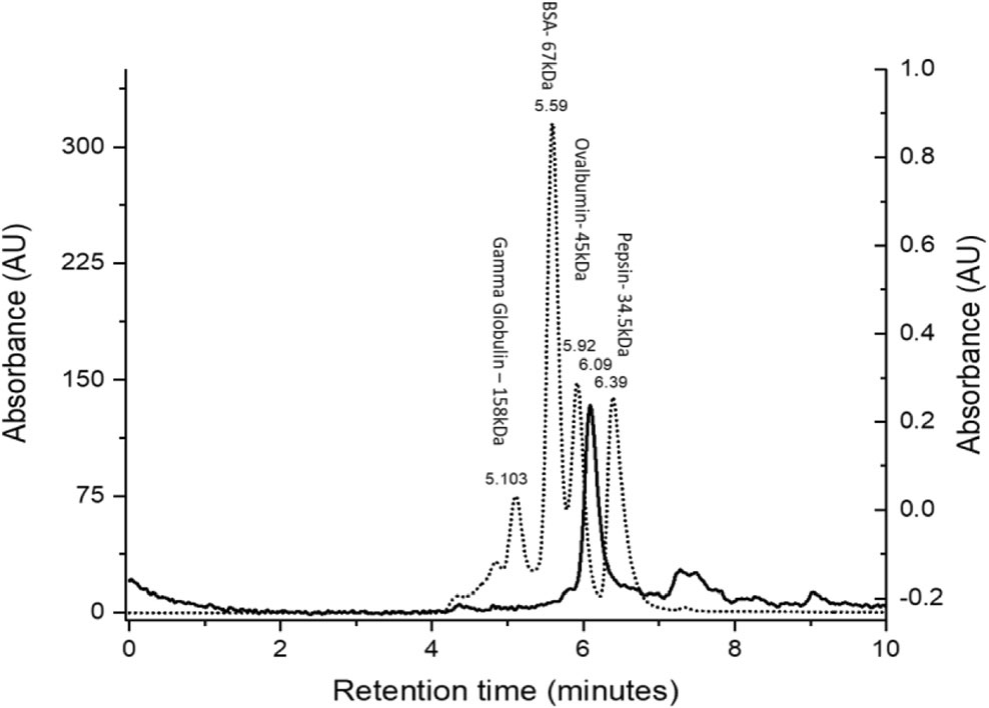
Size exlcusion chromatography (SEC) results of RpaL and four standards. The dotted line indicates standards: (1) gamma globulin (158 kDa) RT, 5.103; (2) BSA (67 kDa) RT, 5.59; (3) ovalbumin RT, 5.92 (45 kDa); and (4) pepsin RT, 6.39 (34.5 kDa). The solid line indicates RpaL, which had a retention time of 6.09 min

SEC was used to measure the size of purified RpaL against four standard proteins. RpaL was found to have a retention time of 6.09 min, indicating its size to be between 35 and 45 kDa, which would suggest its native biological conformation is a monomer (Fig. 6). This differs with other members of the TesB-like subfamily where for example TesB, a medium-chain acyl-CoA TE II from *E. coli* (1C8U), forms a dimer of double hotdog domains (Pidugu et al. 2009).

**Fig. 7:**
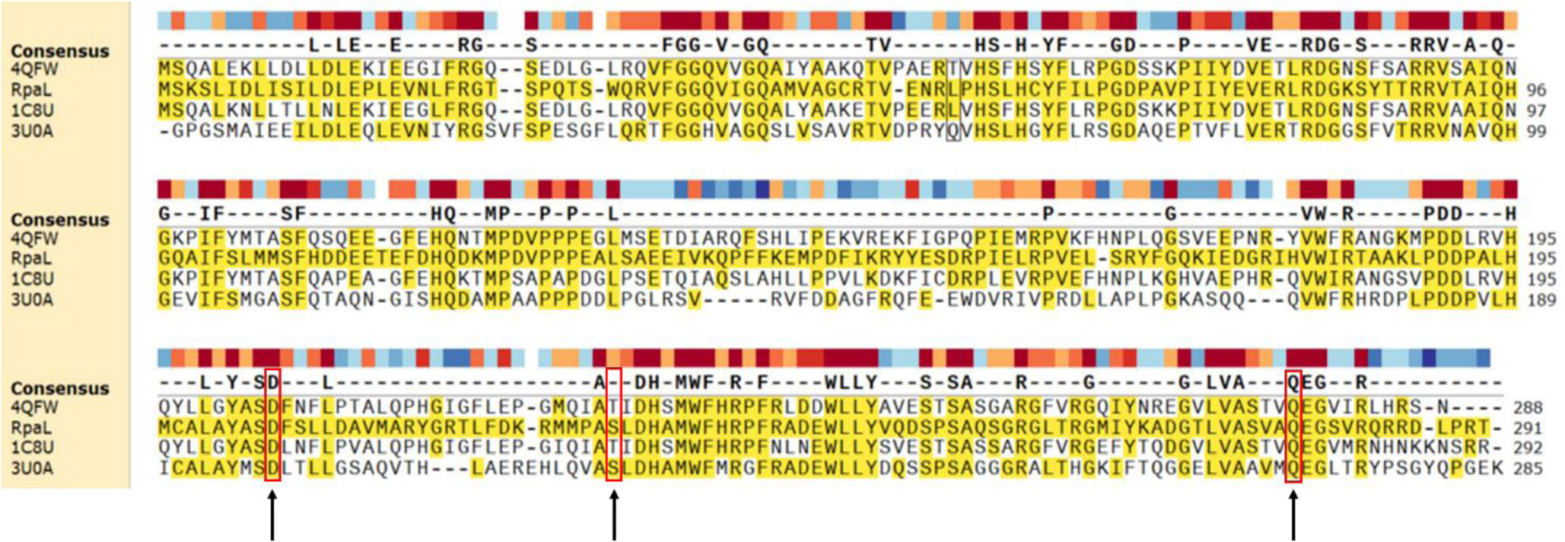
Sequence alignments of RpaL with three TesB-like subfamily TEs, which have the highest sequence similarly of TEs with resolved crystal structures. 4QFW, *Yersinia pestis*; 3U0A, *Mycobacterium marinum*; 1C8U, *Escherichia coli*; and RpaL*, Rhodopseudomonas pseudopalustris*. Arrows indicate the conserved catalytic triad residues. Coloured blocks indicate sequence conservation where darkening red specifies high conservation and darkening blue specifies low conservation

Sequence analysis using the RCSB search tool showed the most similar sequence (46% identity) to be an acyl-CoA TesB-like TE from *Yersinia pestis* (4QFW), which forms a homotetramer of double hotdogs. Furthermore, the acyl-CoA TE II TesB2 from *Mycobacterium marinum* (3U0A), which had 45% identity to RpaL, has been shown to form a back-to-back dimer of double hotdog monomers. Despite the variation in the quaternary structures of these four TEs, a sequence alignment shows they have a conserved catalytic triad of Asp204, Ser/Thr228 and Gln278 (Fig. 7).

### Steady-state kinetics of RpaL

**Fig. 8:**
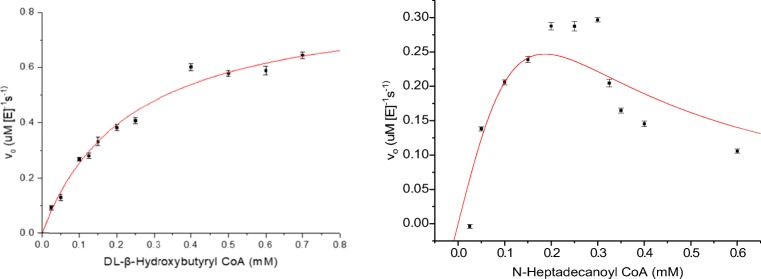
Michaelis–Menten graphs of **a** DL-β hydroxybutyryl CoA, which was the substrate with the highest specificity constant (1.6 × 10^4^ M^−1^ s^−1^) and **b***n*-heptadecanoyl, which showed exaggerated substrate inhibition compared to that predicted from the modified Michaelis–Menten equation

The highest *K*_cat_/*K*_m_ (1.6 × 10^4^ M^−1^ s^−1^) was found with DLBH CoA, with a similarly high-specificity constant with *i*But CoA (1.4 × 10^4^ M^−1^ s^−1^), suggesting a preference for short-branched chain CoA esters (Table 1). However, a similarly high catalytic efficiency (1.2 × 10^4^ M^−1^ s^−1^) was observed with Bz CoA, implying this high catalytic activity can occur on a range of structurally unrelated substrates. Slightly lower catalytic efficiencies were observed with *i*Val CoA and HMG CoA, suggesting that increasing the complexity of the CoA ester has a negative effect on enzyme activity. However, the lowest calculated *K*_cat_/*K*_*m*_, which was found with HMG CoA (4.2 × 10^3^ M^−1^ s^−1^), was still found to be ~ 26% of the highest substrate, DLBH CoA. When determining the steady state kinetics of RpaL with *n*HD CoA, substrate inhibition was observed, with *V*_*0*_ values increasing up to a substrate concentration of 0.3 mM, which was followed by a sharp *V*_*0*_ decline. When attempting to fit the calculated *V*_*0*_ values with the modified Michaelis–Menten equation, *V*0 = *V* max [*S*]/(*Km* + [*S*](1 + [*S*]/*Ki*), a much steeper decrease in *V*_*0*_ was observed than expected (Fig. 8).

**Table 1:** Steady-state kinetic parameters with standard error for purified RpaL with CoA esters

Substrate	*K*_*m*_ (mM)	*V*_max_ (μMs^−1^)	*K*_cat_ (s^−1^)	*K*_cat_/*K*_m_(M^−1^ s^−1^)
Isobutyryl CoA (*i*But CoA)	0.36 ± 0.16	1.22 ± 0.25	5.1 ± 1	1.4 × 10^4^
Isovaleryl CoA (*i*Val CoA)	0.11 ± 0.02	0.12 ± 0.01	0.51 ± 0.03	4.6 × 10^3^
DL-β-hydroxybutyryl CoA (DLBH CoA)	0.24 ± 0.03	0.86 ± 0.04	3.74 ± 0.19	1.6 × 10^4^
β-Hydroxy β-methylglutaryl CoA (HMG CoA)	0.2 ± 0.05	0.2 ± 0.02	0.84 ± 0.1	4.2 × 10^3^
Benzoyl CoA (Bz CoA)	0.18 ± 0.02	0.5 ± 0.02	2.08 ± 0.07	1.2 × 10^4^

## Discussion

### Size and biological conformation

The basic subunits (monomers) of a hotdog-fold TE are 5–6 antiparallel beta sheets that are wrapped around an α-helix. These monomers dimerise, forming the basic structural repeat unit (homodimer) of a hotdog-fold protein, through interactions between their beta sheets leading to a continuous beta sheet and two antiparallel alpha helices (Pidugu et al. 2009). This structure is found in FcbC, PA2801 and YbdB, which all form tetramer quaternary structures, with considerable variation with how they each dimerise. FcbC and YbdB dimerise through association of their beta sheets (back-to-back association), whereas PA2801 dimerises through alpha helix associations (face-to-face) (Thoden et al. 2003; Gonzalez et al. 2012; Wu et al. 2014). In addition to homodimers, the other observed basic structural repeat unit is a polynucleotide fusion of two hotdog domains, which upon translation forms two hotdog domains. This is the case for RpaL which is shown to be ~ 36 kDa, twice the size of the monomers of the other expressed TEs (Fig. 2). Despite knowing the size of the linear polypeptide chain, the native biological configuration (i.e. monomer, dimer and tretramer) of RpaL was unknown. Previous investigations looking into the biophysical properties of members of the TE4 family (TesB-like subfamily) containing a double hotdog domain have shown complex tetrameric structures to be formed (Pidugu et al. 2009; Swarbrick et al. 2015). SEC was used to determine the native size of RpaL, which showed a single protein elution corresponding to a biologically active size of ~ 36 kDa, consistent with a monomer. This data may suggest that RpaL does not need to form complex tetrameric quaternary structures for viable activity.

### Aberrant activity with NAC esters

Due to the high cost and limited commercial availability of CoA esters, previous investigations have used *N*-acetylcysteamine (NAC) thioesters for screening TE activity. These thioesters contain the first part of the 4′-phosphopantetheine arm (Fig. 3a and b) of the CoA, and so act as a synthetic mimic of a CoA. Several investigations have shown NAC esters to be viable mimics of their CoA ester counterparts for TEs from the α/β-hydrolase-fold superfamily (Korman et al. 2010). However, there has been limited work assessing whether TEs from the hotdog-fold superfamily can also use NAC esters as viable mimics. To gain insight, this study screened each TE with cyclohexanecarbonyl-NAC (CHC-NAC) as well as cyclohexanecarbonyl-CoA (CHC CoA). Interestingly, the two TEs which were able to hydrolyse CHC CoA (PA2801 and RpaL) were unable to hydrolyse CHC-NAC, highlighting a stringent requirement for the entire CoA component, despite a promiscuous acceptance of CoA-activated organic acids.

Investigations looking into the structure and catalytic mechanism of hotdog-fold TEs have shown there to be a conserved nucleotide-binding site on the enzyme surface that binds to the 3′-phosphate (P) and 5′-pyrophosphate (PP) of the nucleotide moiety of the CoA (Song et al. 2012; Wu et al. 2014). In FcbC, the CoA 3’-P forms ions pairs with the side chains of Arg102 and Arg150 and the 5’-PP hydrogen bonds with Ser120 and Thr121. It was shown that the mutants FcbC T121A, R150A and R102A all resulted in significantly decreased *k*_cat_/*K*_*m*_, highlighting the importance of these interactions (Song et al. 2012). As the NAC esters only mimic the first portion of the 4′-phosphopantetheine arm (Fi50p g. 3), none of these interactions between the surface of the protein and CoA nucleotide can occur. As such, this may explain why both PA2801 and RpaL could use CHC CoA as a substrate but not CHC-NAC. Furthermore, with only a few exceptions, members of the α/β-hydrolase-fold family predominantly target substrates bound to acyl carrier proteins (ACP) or peptidyl carrier proteins (PCP) (Cantu et al. 2010). Here, substrates are covalently bound to a 4′-phosphopantetheine (PPT) arm, which lacks the nucleotide moiety of a CoA. This may suggest that interactions between the TE surface and substrate are not critical for hydrolysis, possibly explaining why NAC thioesters can be used as viable substrate mimics with α/β-hydrolase-fold TEs and not hotdog-fold TEs.

### Steady-state kinetics

RpaL was shown to be able to hydrolyse a variety of CoA esters including aromatics, short- and long-chain aliphatics and alicyclics. This ability to hydrolyse such a diverse range of CoA esters, with a high-specificity constant, makes it an extremely desirable candidate for the ‘Termination’ step of the proposed organic acid modification system. By implementing such a promiscuous TE within the termination step would mean that a plethora of CoA esters could be produced in the preceding ‘activation’ and ‘modification’ steps, which could all be hydrolysed by a single-enzyme RpaL, without the need for identifying a separate TE for each CoA ester.

RpaL was able to hydrolyse the long-chain fatty acyl-N-heptadecanoyl CoA, at concentrations up to 0.3 mM, above which a dramatic decline in *V*_*0*_ was observed that could not be fitted to the modified Michaelis–Menten equation. This may be a result of more complex substrate inhibition than can be modelled using the standard substrate model. As substrate inhibition was not observed with any of the other CoA esters, it is also possibly due to the long acyl chain. In previous investigations that have screened hotdog-fold TEs with fatty acyl-CoA esters, substrate inhibition has also been observed with longer chain acyl-CoAs such as palmitoyl CoA and oleoyl CoA (Wei et al. 2009). Here, it was suggested that micelle formation may result in apparent substrate inhibition, as it was shown that the *V*_*0*_ of both substrates increased up to the critical micellar concentration (CMC), after which point the *V*_*0*_ declined. It is possible that a similar effect has occurred in the current investigation and provides an explanation as to why a modified Michaelis–Menten equation does not fit the observed *V*_*0*_ data.

In the work presented here, four hotdog-fold TEs were cloned, expressed and screened with a range of aliphatic, alicyclic and aromatic CoA esters, as well as a synthetic CoA mimic, *N*-acetylcysteamine (NAC). Three of the four TEs showed relatively narrow substrate specificity; however, RpaL from *Rhodopseudomonas palustris* (HaA2) was shown to have extremely promiscuous activity. PA2801 and RpaL, two TEs that could hydrolyse CHC CoA, were unable to use the synthetic mimic CHC-NAC, which may be due to absent ion pairs and hydrogen bonding between the TE and NAC component of the NAC ester.

Due to its promiscuous substrate acceptance, RpaL was purified for determination of steady-state kinetic constants. SEC showed RpaL to be a biologically active monomer with no clear complex quaternary structures, unlike the complex tetrameric quaternary structures observed in other characterised members of TesB-like subfamily (TE4). However, despite the structural diversity of members of the TesB-like subfamily of TEs, they share conserved catalytic residues. Substrate saturation curves fitted with the Michaelis–Menten equation determined the highest specificity constant to be towards DLBH CoA, with decreasing specificity constants observed with substrates containing increased functional complexity.

With the identification of a TE that is able to utilise such a diverse range of CoA esters with high activity, it is an ideal candidate for implementation in the final step (termination) of the proposed CoA ester-modifying system (Scheme 1). By having such broad activity, it will provide flexibility to a CoA ester modification system allowing any type of CoA ester produced to be hydrolysed into its corresponding organic acid. Ongoing research is focused on developing a promiscuous CoA ligase as well as building a toolbox of CoA-dependent enzymes to be used in the activation and modification components of the system, respectively. Once completed, each of these modules will be combined resulting in a flexible biosynthetic system that will be capable of producing a range of complex organic acids, expanding the portfolio of specialty chemical synthons and proving a unique approach for increasing the design space of novel organic acids.

## Electronic supplementary material

ESM 1 (PDF 766 kb)
