# Supplementary material for: Characterisation of four hotdog-fold thioesterases for their implementation in a novel organic acid production system
Source: Appl Microbiol Biotechnol. 2020 Mar 19;104(10):4397–406. doi: 10.1007/s00253-020-10519-w (PMC7190597; doi:10.1007/s00253-020-10519-w)
Supplement: Supplementary file 1 — (PDF 766 kb) [file 253_2020_10519_MOESM1_ESM.pdf]

Journal: Applied Microbiology and Biotechnology

Characterisation of four Hotdog fold Thioesterases for their implementation in a novel organic acid production system

Hickman T. W. P.,[a] Baud D.,[b] Benhamou L.,[b] Hailes H. C.,[b] Ward J. M.*,[a]

[a]Department of Biochemical Engineering, University College London, Gower Street, London, WC1E 6BT, UK

[b]Department of Chemistry, University College London, 20 Gordon Street, London, WC1H 0AJ, UK

*Corresponding author:
e-mail: j.ward@ucl.ac.uk,
Tel: 020 76799568

Supplementary material

Chemical synthesis

All chemicals were obtained from commercial suppliers and used as received. Thin layer chromatography was carried out using Merck TLC Silica gel 60 F254 plates and products were visualised using combinations of UV light (254 nm) and potassium permanganate staining solutions. Filtrations and silica chromatography were carried out using silica gel (particle size 40–60 μm).

^{1}H NMR spectra were recorded at 300 MHz, on a Bruker AMX300 MHz spectrometer using the residual protic solvent stated as the internal standard. Chemical shifts are quoted in ppm to the nearest 0.01 ppm using the following abbreviations: s (singlet), d (doublet), t (triplet), q (quartet), or combinations thereof. ^{13}C{^{1}H} NMR spectra were recorded at 75 MHz on a a Bruker AMX300 MHz at 25 °C using the solvent stated. Chemical shifts are reported to the nearest 0.1 ppm. The coupling constants are defined as *J* and quoted in Hz. Mass spectra were performed in the Department of Chemistry (University College London).

S-(2-Acetamidoethyl) cyclohexanecarbothioate CHC NAC

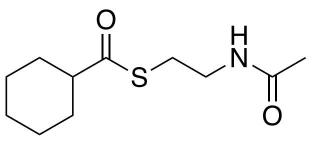

To cyclohexanecarboxylic acid (160 mg, 1.25 mmol) and *N*-acetyl cycteamine (196 μL, 1.85 mmol) in dichloromethane (5 mL) at 0 °C, *N*,*N*′-dicyclohexylcarbodiimide (DCC) (281 mg, 1.36 mmol) and dimethylaminopyridine (DMAP) (30 mg, 0.25 mmol) were added. The reaction mixture was warmed to room temperature and stirred for 4 h. The reaction was then filtered through silica and concentrated *in vacuo*. The crude product was purified using silica column chromatography (CH_2Cl_2/MeOH, 97:3) to afford CHC NAC (140 mg, 49%) (1).

FIG S1. ^{1}H NMR spectrum**.** ^{1}H NMR (300 MHz, $CDCl_3$) □ 6.04 (1H, br s), 3.27 (2H, app. q, *J* 6.5 Hz, CH_2N), 2.87 (2H, t, *J* 6.5 Hz, CH_2S), 2.31-2.40 (1H, tt, *J* 11.3, 3.5 Hz, CHCO), 1.86 (3H, s, CH_3CO), 1.85-1.62 (4H, m), 1.61-1.47 (1H, m), 1.46-1.25 (2H, m), 1.25-0.90 (3H, m); ^{13}C NMR (75 MHz, $CDCl_3$) □ 203.3 (SC=O), 170.4, HNC=O), 52.4, 39.3, 29.3, 27.7, 25.3, 25.2, 22.9.

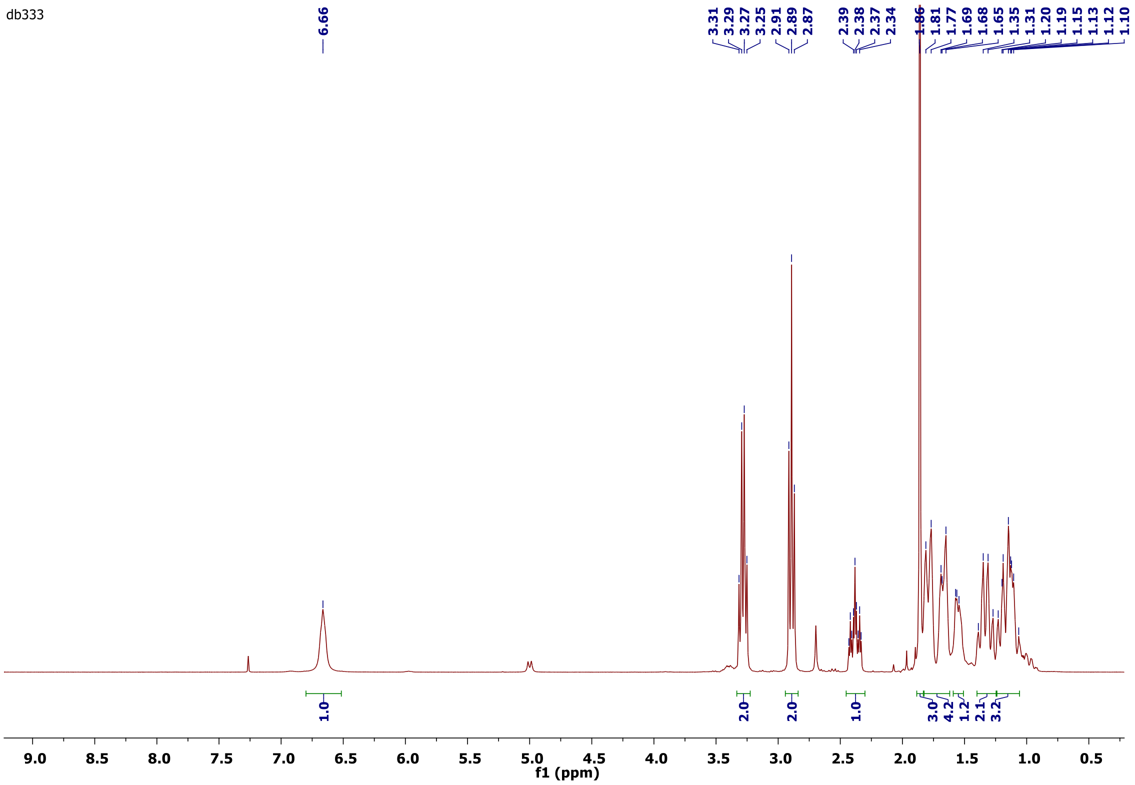

FIG S2. ^{13}C NMR spectrum

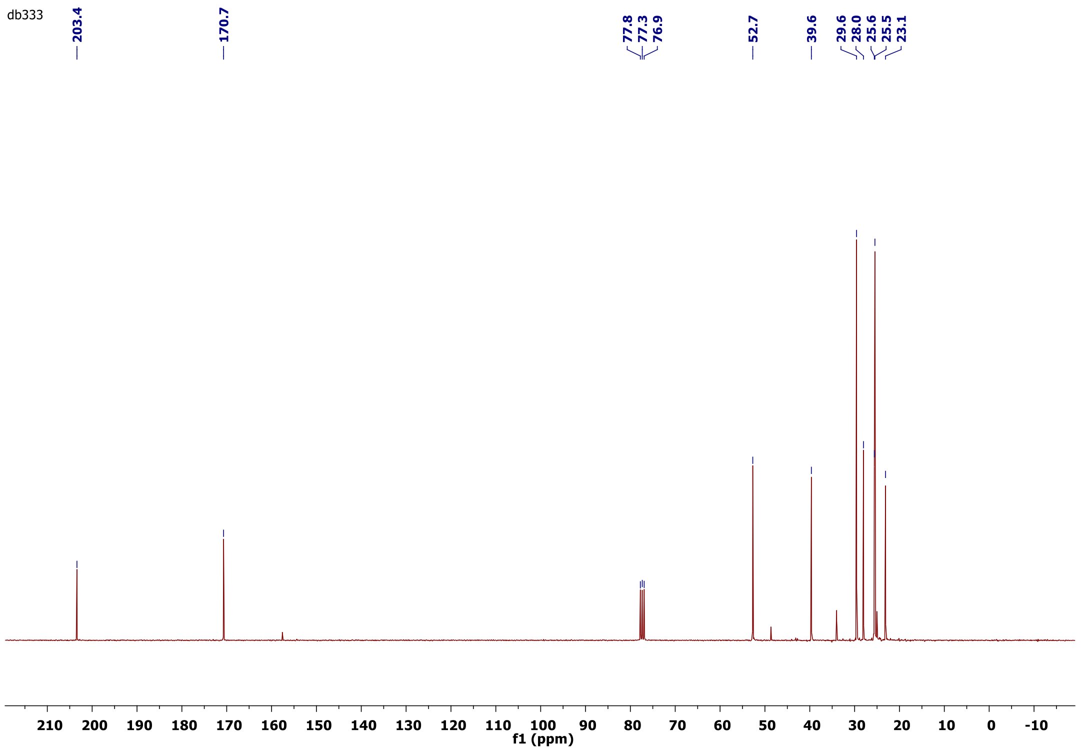

CHC CoA

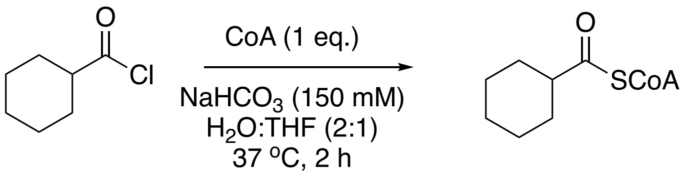

Cyclohexanecarbonyl chloride (3.4 µL, 25 µmol) was added to a solution of coenzyme A (10 mg, 13 µmol,) in a mixture of THF:H_2O (1.2 mL, 1:2) containing $NaHCO_3$ (150 mM). The resulting mixture was stirred at 37 °C for 2 hours before evaporation of the volatiles under reduced pressure. The white solid formed was analysed by LC-MS, confirming that the CoA derivative was formed, and was used without further purification. *m/z* [ES^-]: 876.5 $[M-H]^-$, 437.8 $[M-2H]^{2-}$.

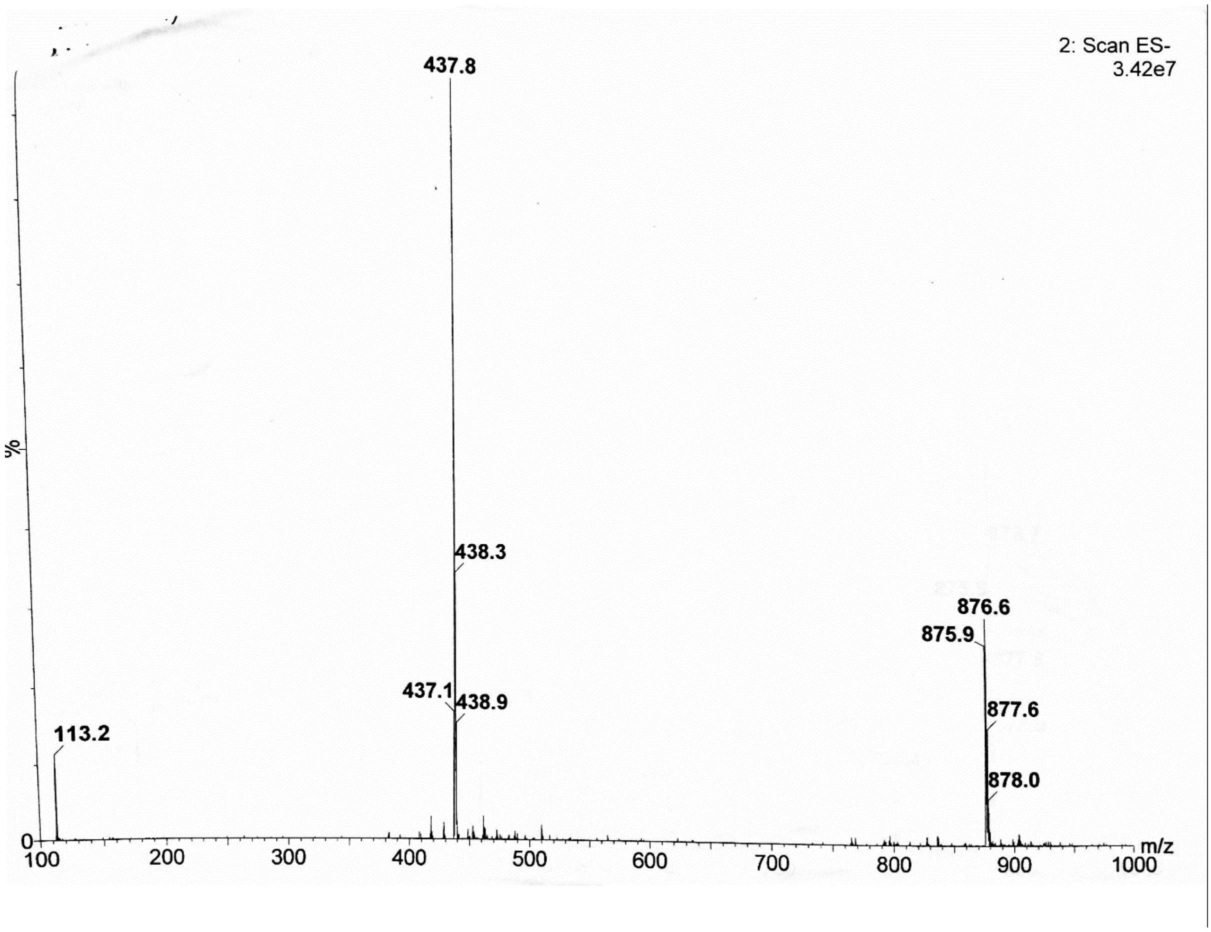

FIG S3. LC-MS analysis confirming that the CoA derivative (CHC CoA) was formed.

FcbC

The nucleotide sequence was derived by reverse translating the 4-hydroxybenzoyl CoA TE (accession number: Q04416) and codon optimising it for expression in *E. coli* using the reverse translate tool in the sequence manipulation suite available at: http://www.bioinformatics.org/sms2/rev_trans.html.

ATGCATCGCACTTCGAATGGTTCTCACGCAACTGGTGGTAATTTGCCTGACGTCGCTAGCCACTAC

CCGGTCGCCTACGAGCAGACGCTGGATGGTACCGTTGGCTTTGTGATTGACGAAATGACCCCGGA

GCGTGCAACCGCGTCCGTCGAGGTTACGGATACGCTGCGTCAACGCTGGGGTCTGGTCCACGGTG
GCGCGTATTGTGCGCTGGCAGAAATGCTGGCGACCGAAGCAACCGTGGCCGTTGTTCATGAGAAA
GGCATGATGGCCGTGGGCCAGAGCAACCATACCAGCTTTTTCCGTCCGGTGAAGGAAGGCCACGT
GCGCGCAGAGGCTGTGCGCATCCACGCGGGCAGCACGACCTGGTTCTGGGATGTTAGCCTGCGTG
ACGACGCCGGTCGTCTGTGCGCGGTCAGCTCCATGAGCATCGCGGTTCGTCCGCGTCGTGATGGTG
GTAGCCACCACCACCATCATCATTAATGA

Amino acid sequence:

MHRTSNGSHATGGNLPDVASHYPVAYEQTLDGTVGFVIDEMTPERATASVEVTDTLRQRWGLVHGG
AYCALAEMLATEATVAVVHEKGMMAVGQSNHTSFFRPVKEGHVRAEAVRIHAGSTTWFWDVSLRD
DAGRLCAVSSMSIAVRPRRDGGSHHHHHH

PA2801

For primer design and cloning of PA2801 the nucleotide sequence (Accession number: AAG06189) was used.

ATGGCTGACAGACAATTGCTACACACGGCCCATATCCCGGTGCGCTGGGGCGACATGGACAGCTA
CGGGCACGTCAACAACACCCTCTATTTCCAGTACCTGGAAGAGGCCCGGGTGGCCTGGTTCGAAA
CCCTCGGCATCGACCTGGAAGGCGCTGCCGAGGGGCCGGTCGTGCTGCAAAGCCTGCACACCTAC
CTGAAGCCGGTGGTGCATCCGGCCACCGTGGTGGTGGAACTGTACGCCGGCAGGCTGGGCACCAG
CAGCCTGGTACTGGAACATCGCCTGCACACCCTGGAAGATCCGCAAGGCACCTATGGCGAAGGCC
ACTGCAAGCTGGTCTGGGTGCGCCACGCGGAAAACCGTTCGACGCCCGTGCCGGACAGCATACGC
GCCGCGATCGCCTGA

Amino acid sequence:

MADRQLLHTAHIPVRWGDMDSYGHVNNTLYFQYLEEARVAWFETLGIDLEGAAEGPVVLQSLHTYL
KPVVHPATVVVELYAGRLGTSSLVLEHRLHTLEDPQGTYGEGHCKLVWVRHAENRSTPVPDSIRAAIA

RpaL

The nucleotide sequence for RpaL was derived from *Rhodopseudomonas palustris* HaA2 accession number ABD05081 and codon optimised *for* expression in *E. coli*.

ATGGGCAGCAGCCATCATCATCATCATCACAGCAGCGGCCTGGTGCCGCGCGGCAGCCATATGAG
CAAAAGCCTGATTGATCTGATTAGCATTCTGGATCTGGAACCGCTGGAAGTGAACCTGTTTCGCGG
CACCAGCCCGCAGACCAGCTGGCAGCGCGTGTTTGGCGGCCAGGTGATTGGCCAGGCGATGGTGG
CGGGCTGCCGCACCGTGGAAAACCGCCTGCCGCATAGCCTGCATTGCTATTTTATTCTGCCGGGCG
ATCCGGCGGTGCCGATTATTTATGAAGTGGAACGCCTGCGCGATGGCAAAAGCTATACCACCCGC
CGCGTGACCGCGATTCAGCATGGCCAGGCGATTTTTAGCCTGATGATGAGCTTTCATGATGATGAA
GAAACCGAATTTGATCATCAGGATAAAATGCCGGATGTGCCGCCGCCGGAAGCGCTGAGCGCGGA
AGAAATTGTGAAACAGCCGTTTTTTAAAGAAATGCCGGATTTTATTAAACGCTATTATGAAAGCGA
TCGCCCGATTGAACTGCGCCCGGTGGAACTGAGCCGCTATTTTGGCCAGAAAATTGAAGATGGCC
GCATTCATGTGTGGATTCGCACCGCGGCGAAACTGCCGGATGATCCGGCGCTGCACATGTGCGCGC
TGGCGTATGCGAGCGATTTTAGCCTGCTGGATGCGGTGATGGCGCGCTATGGCCGCACCCTGTTTG
ATAAACGCATGATGCCGGCGAGCCTGGATCATGCGATGTGGTTTCATCGCCCGTTTCGCGCGGATG
AATGGCTGCTGTATGTGCAGGATAGCCCGAGCGCGCAGAGCGGCCGCGGCCTGACCCGCGGCATG
ATTTATAAAGCGGATGGCACCCTGGTGGCGAGCGTGGCGCAGGAAGGCAGCGTGCGCCAGCGCCG
CGATCTGCCGCGCACCTAATAG

Amino acid sequence:

MGSSHHHHHHSSGLVPRGSHMSKSLIDLISILDLEPLEVNLFRGTSPQTSWQRVFGGQVIGQAMVAGCR
TVENRLPHSLHCYFILPGDPAVPIIYEVERLRDGKSYTTRRVTAIQHGQAIFSLMMSFHDDEETEFDHQD
KMPDVPPPEALSAEEIVKQPFFKEMPDFIKRYYESDRPIELRPVELSRYFGQKIEDGRIHVWIRTAAKLPD
DPALHMCALAYASDFSLLDAVMARYGRTLFDKRMMPASLDHAMWFHRPFRADEWLLYVQDSPSAQ
SGRGLTRGMIYKADGTLVASVAQEGSVRQRRDLPRT

The nucleotide sequence used for primer design for YbdB was Accession: P0A8Y8.

ATGATCTGGAAACGCCATTTAACGCTCGACGAACTGAACGCCACCAGCGATAACACAATGGTGGC
GCATCTGGGAATTGTGTATACCCGTCTGGGCGATGATGTGCTGGAAGCCGAAATGCCGGTTGATAC
CCGTACTCATCAGCCGTTCGGTTTACTACATGGCGGCGCGTCGGCGGCGCTGGCGGAAACGCTGGG
ATCGATGGCCGGATTTATGATGACCCGCGACGGACAGTGTGTGGTAGGCACAGAACTTAATGCAA
CACACCATCGCCCGGTGTCTGAGGGAAAGGTACGCGGCGTCTGCCAGCCGCTGCATCTTGGTCGGC
AAAATCAGAGCTGGGAAATCGTCGTTTTCGATGAACAGGGGCGGCGTTGCTGCACTTGTCGGCTG
GGTACGGCAGTTTTGGGATGA

Amino acid sequence

MIWKRHLTLDELNATSDNTMVAHLGIVYTRLGDDVLEAEMPVDTRTHQPFGLLHGGASAALAETLGS
MAGFMMTRDGQCVVGTELNATHHRPVSEGKVRGVCQPLHLGRQNQSWEIVVFDEQGRRCCTCRLGT
AVLG

References:

1. Prasad G, Borketey LS, Lin TY, Schnarr NA. 2012. A mechanism-based fluorescence transfer assay for examining ketosynthase selectivity. Org Biomol Chem.
